# A Randomized Comparative Trial on the Therapeutic Efficacy of Topical *Aloe vera* and *Calendula officinalis* on Diaper Dermatitis in Children

**DOI:** 10.1100/2012/810234

**Published:** 2012-04-19

**Authors:** Yunes Panahi, Mohamad Reza Sharif, Alireza Sharif, Fatemeh Beiraghdar, Zahra Zahiri, Golnoush Amirchoopani, Eisa Tahmasbpour Marzony, Amirhossein Sahebkar

**Affiliations:** ^1^Chemical Injuries Research Center, Baqiyatallah University of Medical Sciences, P.O. Box 19945-581, Tehran, Iran; ^2^Department of Pediatrics, Kashan University of Medical Sciences, P.O. Box 88175-111, Kashan, Iran; ^3^Department of Infectious Diseases, Kashan University of Medical Sciences, P.O. Box 88175-111, Kashan, Iran; ^4^Nephrology and Urology Research Center, Baqiyatallah University of Medical Sciences, P.O. Box 19945-581, Tehran, Iran; ^5^Pharmaceutical Sciences Branch, Islamic Azad University, P.O. Box 19945-581, Tehran, Iran; ^6^Biotechnology Research Center and School of Pharmacy, Mashhad University of Medical Sciences (MUMS), P.O. Box 91775-1365, Mashhad, Iran

## Abstract

*Introduction*. Diaper dermatitis (DD) is a common inflammatory disorder among children and infants. The objective of the present randomized and double-blind trial was to compare the therapeutic efficacies of *Aloe vera* cream and *Calendula officinalis* ointment on the frequency and severity of DD in children. *Methods*. Sixty-six infants with DD (aged < 3 years) were randomized to receive either *Aloe* cream (*n* = 32) or *Calendula* ointment (*n* = 34). Infants were treated with these drugs 3 times a day for 10 days. The severity of dermatitis was graded at baseline as well as at the end of trial using a 5-point scale. The adverse effects of study medications were assessed during the trial. *Results*. Although improvement in the severity of DD was observed in both treatment groups (*P* < 0.001), patients receiving *Calendula* ointment had significantly fewer rash sites compared to *Aloe* group (*P* = 0.001). No adverse effect was reported from either of the medications. *Discussion*. The evidence from this study suggests that topical *Aloe* and in particular *Calendula* could serve as safe and effective treatment for the treatment of diaper dermatitis in infants.

## 1. Introduction

Diaper rash or diaper dermatitis (DD) is a common type of dermatitis among the infants and children who wear diapers. It refers to any acute inflammatory skin eruption that occurs in area covered by diaper and caused by either direct effect of wearing diapers or as a result of increased skin pH, zinc deficiency, prolonged exposure to moisture, and irritants like urine and feces [1–3]. The combination of these factors leads to overhydration of the stratum corneum as well as chemical and mechanical abrasion, which compromises barrier function and makes the stratum corneum more susceptible to frictional trauma and the penetration of irritants and microbes [4, 5]. In addition, the presence of microorganisms especially *Candida* plays a secondary role in the development of DD [2, 6]. DD is uncommon during the first few months of life as fecal enzymes are present in low levels during this period. It usually peaks between 6 and 12 months of age and may continue till diapers are not further used in children. Because of some negative side effects such as irritation, erythema, and papules, it is essential to identify effective strategies in order to decrease the prevalence of DD in children or infants, particularly if a patient does not respond to standard therapy.

The management of DD should include reducing moisture in the diaper area, minimizing contact with urine and feces, and eradicating infectious microorganisms. Diaper technology has improved significantly over the last few decades and continues to evolve. For example, disposable diapers that contain superabsorbent gelling materials or zinc oxide/petrolatum formulation are associated with a reduced incidence and decreased severity of DD [7, 8]. Moreover, numerous products such as petrolatum, zinc oxide, corticosteroids, vitamins A and D, and lanolin are available for the treatment of uncomplicated DD [1, 9]. Although many infants may benefit from these products, the healthcare provider must be aware of children who are allergic to some of these products. In addition, these products are more effective only for the treatment of moderate DD [1]. Therefore, clinicians should become familiar with the benefits and drawbacks of the many products available for the treatment of DD.

The use of medicinal plants as antibacterial and anti-inflammatory drugs in folk medicine is a practice common in Iran [10]. *A. vera *and* C. officinalis* are two medicinal plants with diverse biological activities including anti-inflammatory and antimicrobial effects [11–18]. Since the incidence of DD among children and infants is high and they may have allergy to some chemical drugs, the present trial aimed to evaluate the efficacy of *A. vera *cream and *C. officinalis* ointment in the alleviation of DD symptoms. The secondary goal was to compare the effects of these two natural drugs.

## 2. Materials and Methods

This study was conducted from August 2010 to September 2011 in Dermatology Clinic (PNICU) at the Baqiyatallah Hospital (Tehran, Iran). After obtaining institutional review board approval and informed consent, we studied 66 children with DD (aged < 3 years old, 32 females). The planned study duration was 10 days. Patients who had secondary infections and treated with corticosteroids or had sensitivity to any of the study medications were excluded from participation.

Recruited children were randomized to receive topical *Aloe *(*n* = 32) or *Calendula* (*n* = 34). *Calendula* ointment (Dineh Iran Pharmaceutical Co., Tehran, Iran) contained 1.5% of total extract obtained from *C. officinalis* flowers. *Aloe* cream (Kia Behdasht Pharmaceutical Co., Hashtgerd, Iran) contained* A. vera* gel and olive oil as active ingredients which were prepared in an oil/water emulsion base. The components of the emulsion cream base were stearic acid, cetyl alcohol, Vaseline, mineral oil, glycerol monostearate, glycerin, propylene glycol, triethanolamine, methylparaben, propylparaben, and deionized water. The *A. vera*/olive oil ratio in the cream was ~3/2.

Children in each group were treated with the respective topical three times a day for a period of 10 days. Parents were instructed to wash diaper area with lukewarm water during diaper change and—after drying—treat the area only with the administered medication. The severity of dermatitis was graded at baseline as well as days 5 and 10 of study using a 5-point scale (from 0 to 4) according to Davis et al. [19]. The zero score was representative of no erythema, while 1–4 scores were indicative of mild erythema with minimal maceration and/or chafing; moderate erythema with or without satellite papules with maceration and chafing; severe erythema with papulopustules and maceration; extreme erythema with erosions or ulceration, respectively.

After the 5th day of examination, the treatment was stopped for patients who had received complete health by that time, but it was continued to the 10th day for children who were not completely recovered. The episodes of any adverse effects throughout the study period were also assessed. 

Statistical analyses were performed using Statistical Package for Social Sciences (SPSS), version 16. Data were expressed as mean ± SD or number (%). Group comparisons were made using Mann-Whitney *U* test, Wilcoxon-signed rank test, Student's *t-*test, or one-way analysis of variance (ANOVA). Categorical variables were compared using chi-square or Fisher's exact test. A *P*-value of less than 0.05 was considered to be statistically significant.

## 3. Results

There was no statistically significant difference between the groups regarding age, gender, and daily frequencies of diaper change and washing diaper area. Demographic characteristics of the study groups are summarized in Table 1.

Table 2 indicates data regarding the past history of diseases that might influence the development of DD. These diseases included diarrhea, immunodeficiency, renal deficiency, powdered milk, food or drug hypersensitivity, hematochezia, chest wheezing, facial eczema, oral thrush, and anemia. No significant difference in the prevalence of the aforementioned disorders was observed between the groups (*P* > 0.05).

Table 3 depicts the prevalence of DD severity in both groups before and after study. Although the severity of DD was clearly decreased in both groups by the end of trial (*P* < 0.001), the reduction rate was found to be significantly greater in the *Calendula* group (*P* = 0.001). There was not any adverse effect from either of the study drugs.

## 4. Discussion

Diaper rash is of considerable importance to parents, pediatricians, and other caregivers. While it is generally thought to affect infants and toddlers, any individual wearing a diaper is a candidate to develop this type of dermatitis. It is a common problem that accounts for frequent visits to the pediatrician each year and causes concern to families [20]. In a recent UK study that included the parents of 532 hospitalized children in diapers, 52% of families reported a history of DD and multivariate analysis demonstrated the risk of DD to be associated with oral thrush, past history of disease, frequency of diaper changes, and diarrhea [1, 21]. Therefore, an appropriate approach for diagnosis and treatment of DD is essential. In addition, clinicians should become familiar with the advantages and disadvantages of the many products available for the treatment of DD. To date, numerous products such as petrolatum, zinc oxide, corticosteroids, talcum powder, vitamins A and D, and lanolin have become available for the treatment of uncomplicated DD [1, 9]. Although many infants may benefit from these products, some may be allergic to them. In addition, some of these products may not be effective for moderate-to-severe DD and may have side effects at high concentrations. For the treatment of mild DD, a petrolatum product, vitamins A and D, or a product containing 10% zinc oxide may be sufficient, but for moderate-to-severe DD, a barrier ointment with a greater concentration of zinc oxide is usually required [1]. Zinc oxide pastes are highly effective barriers, but they are difficult to wash off, and aggressive cleansing of the skin when removing a paste is very irritating. The use of talcum powder has also been associated with severe respiratory distress caused by accidental inhalation [22]. Corticosteroids are generally contraindicated for use in intertriginous and occluded areas of the skin, especially in infants, as their use has the potential to cause a variety of adverse events such as systemic absorption, skin atrophy, and growth delay [23]. Thus, for more severe forms of DD, proper diagnosis and treatment is essential

Plant-derived products have been extensively used in the traditional medicine. Recently, researchers have studied the anti-inflammatory effects of herbal drugs for the treatment of a number of inflammatory diseases such as dermatitis. Al-Waili [24] studied the therapeutic effect of a cream, made by combination of honey, olive oil, and beeswax, on 12 children (aged from 3 to 18 month) with DD. Children were treated with cream 4 times a day for 7 days. After day 7, the severity of DD was significantly declined in most patients. In another study, the therapeutic effect of olive oil was evaluated on 173 children with DD [25]. Patients who were treated with olive oil showed significantly lower frequency of diaper rash compared with the control group. Vardy et al. [26] studied the effect of *A. vera* extract on 44 patients with seborrheic dermatitis. It was concluded that irritation and cutaneous scaling were significantly decreased in children who were treated with *A. vera*. In this research, we studied the therapeutic impact of topical *C. officinalis* and *A. vera* on the frequency and severity of DD. Although improvement of rash from baseline was observed in both treatment groups, *Calendula* ointment had higher therapeutic effect compared to *Aloe *cream. These beneficial effects could be attributed to the well-known anti-inflammatory and antimicrobial properties of these two medicinal herbs [11–18]. Another important issue that deserves attention is the safety of these herbal products as there was no reported adverse effect in any of the groups. Based on the findings of the present study, the relevance of topical *Calendula* and *Aloe* as natural, effective, and safe treatments for DD is clearly supported. Further research regarding the comparison of these creams with other commercially available products is strongly recommended.

## Figures and Tables

**Table 1:** Demographic characteristics of study groups.

Parameters	*Aloe* (*n* = 32)	*Calendula *(*n* = 34)	*P* value
Gender			
Male	18 (56.3%)	16 (47.1%)	0.47
Female	14 (43.7%)	18 (52.9%)	0.47
Age			
<6 months	4 (12.5%)	8 (25.8%)	0.34
6–12 months	16 (50%)	14 (45.2%)	0.62
12–24 months	9 (28.1%)	6 (19.14%)	0.38
>24 months	3 (9.4%)	3 (9.7%)	1.00
Diaper changing (per day)			
≤3 time	7 (22.6%)	5 (15.2%)	0.53
4 time	17 (54.8%)	21 (63.6%)	0.62
>4 time	7 (22.6%)	7 (21.2%)	1.00
Washing diaper area (per day)			
1 time	6 (18.8%)	4 (11.8%)	0.50
2 time	8 (25%)	8 (23.5%)	1.00
>2 time	18 (56.3%)	22 (64.7%)	0.61

Values are expressed as number (%). Comparisons have been made using chi-square or Fisher's exact test.

**Table 2:** Past medical history of study groups.

Parameters	*Aloe *(*n* = 32)	*Calendula* (*n* = 34)	*P* value
Powdered milk hypersensitivity	1 (3.1%)	0 (0%)	0.48
Immune deficiency	0 (0%)	0 (0%)	1
Coagulopathy	0 (0%)	1 (2.9%)	1
Renal deficiency	0 (0%)	3 (8.8%)	0.24
Drug hypersensitivity	1 (3.1%)	0 (0%)	0.48
Food hypersensitivity	0 (0%)	1 (2.9%)	1
Plant hypersensitivity	1 (3.1%)	0 (0%)	0.48
Hematochezia	0 (0%)	1 (2.9%)	1
Chest wheezing	1 (3.1%)	0 (0%)	0.48
Diarrhea	4 (12.5%)	2 (5.9%)	0.42
Facial eczema	2 (6.3%)	5 (14.7%)	0.26
Oral thrush	2 (6.3%)	5 (14.7%)	0.43
Anemia	2 (6.3%)	8 (23.5%)	0.08

Values are expressed as number (%). Comparisons have been made using chi-square or Fisher's exact test.

**Table 3:** Severity of diaper dermatitis in the study groups before and after treatment.

Parameters	*Aloe *(*n* = 32)	*Calendula* (*n* = 34)
Before treatment		
0	0 (0%)	0 (0%)
1	1 (3.1%)	0 (0%)
2	11 (33.3%)	7 (20.6%)
3	11 (33.3%)	15 (44.1%)
4	9 (28.1%)	12 (35.3%)
After treatment		
0	0 (0%)	7 (20.6%)
1	18 (56.3%)	17 (50%)
2	9 (25%)	10 (29.4%)
3	2 (6.3%)	0 (0%)
4	3 (9.4%)	0 (0%)

Within-group comparison		

	*P* < 0.001	*P* < 0.001

Between-group comparison		

	*P* = 0.001	

Values are expressed as number (%). Comparisons have been made using Mann-Whitney *U* test, Wilcoxon-signed rank test, or Student's *t-*test.
